# Genetic Variation of Growth Traits and Seed Production in a Patagonian Native Pasture in Semiarid Rangelands Under Different Environmental Settings

**DOI:** 10.3390/plants14050736

**Published:** 2025-02-27

**Authors:** Aldana Soledad López, Nicolás Nagahama, Alejandro Aparicio, María Marta Azpilicueta, Verónica Guidalevich, Juan Pablo Angeli, Paula Marchelli

**Affiliations:** 1 Laboratory of Applied Bioprospecting in Plants and Fungi (LaBIAPH), Faculty of Natural and Health Sciences, National University of Patagonia San Juan Bosco, Ruta Nacional 259, Esquel 9200, Chubut, Argentina; nagahama.nicolas@inta.gob.ar (N.N.); angeli.juan@inta.gob.ar (J.P.A.); 2 Consejo Nacional de Investigaciones Científicas y Técnicas (CONICET), CCT Patagonia Sur, Puerto Madryn 9120, Chubut, Argentina; 3 Consejo Nacional de Investigaciones Científicas y Técnicas (CONICET), CCT Patagonia Norte, San Carlos de Bariloche 8400, Rio Negro, Argentina; guidalevich.veronica@inta.gob.ar (V.G.); marchelli.paula@inta.gob.ar (P.M.); 4 Estación Experimental Agroforestal Esquel, Instituto Nacional de Tecnología Agropecuaria (INTA), Esquel, Chacabuco 513, Esquel 9200, Chubut, Argentina; 5 INTA Bariloche—IFAB (INTA-CONICET), Modesta Victoria 4450, Bariloche 8400, Río Negro, Argentina; aparicio.alejandro@inta.gob.ar (A.A.); azpilicueta.maria@inta.gob.ar (M.M.A.)

**Keywords:** multi-environment field tests, forage, seed production, temperate grass, Patagonia

## Abstract

Rangelands play a crucial socioeconomic and environmental role worldwide. In South America, desertification and overgrazing has led to their deterioration and declining productivity. Breeding programs that use native forage species of economic and ecological importance, such as *Festuca pallescens* (St. Yves) Parodi, may provide locally adapted germplasm that enhances productivity without threatening local biodiversity. These programs may even promote the conservation of native species. To this end, we characterized the phenotypic variation of nondestructive variables (growth and reproductive traits) related to forage and seed production during spring and early summer (growth and reproductive periods). Plants from ten populations were grown under common garden conditions in two environmental settings (sites) over two years. By early summer of the second year, most populations maintained a consistent relative performance with higher values for basal diameter, height and synflorescence production at site 2. This suggests more favorable environmental conditions for the species and highlights their potential for enhancing both seed and forage production. The growth and reproductive traits were probably largely influenced by micro-environmental cues (i.e., soil type and moisture), showing predominantly plastic patterns. The populations displaying phenotypic plasticity and above-average values for both traits were selected for further evaluation in breeding programs.

## 1. Introduction

Rangelands are crucial economic and ecological resources in arid and semiarid regions. They support livestock farming and play a vital role in maintaining biodiversity and soil health [1]. However, long-term overgrazing, combined with decreasing precipitation and rising temperatures [2], has degraded many grasslands in South America’s arid and semiarid regions. This degradation has resulted in reduced productivity and significant economic challenges [3,4]. Within this biome, tussock grasses are the primary vegetation contributing to natural forage production and the preservation of soil organic carbon and nitrogen [5,6]. As the most palatable species, these grasses are often overgrazed, leading to their replacement by shrubs or unpalatable species [7,8]. This continuous degradation of grasslands due to overgrazing and changing environmental conditions has adversely affected both species and genetic diversity [9], significantly reducing the genetic variability within natural populations [10]. Assessing the genetic variation of palatable species, particularly those with a broad distribution in semiarid regions, provides essential information for plant breeding programs. These programs aim to enhance sustainability in local agricultural practices while preserving the ecological integrity of rangelands under environmental stressors.

The search for native grasses with agronomic relevant traits that enhance productivity and resilience is promising for domestication and breeding programs. In tussock grasses, the biomass yield depends on vegetative tiller production while seed production relies on reproductive tillers. Both traits vary significantly with environmental conditions, and there is a trade-off between vegetative and reproductive tiller development. Some tillers undergo floral transition to become reproductive, producing seeds [11], while others remain vegetative throughout the year [12]. Seeds of many tussock species are underrepresented in grassland seed banks [13] due to pre-dispersal seed depletion by livestock and post-dispersal seed consumption by granivores [8]. While tussock reproduces both asexually (via tiller division) and sexually (via seeds), seed production is critical for increasing the genetic variation within clonal plant populations and enabling dispersal and recolonization of disturbed areas [14]. Additionally, growth traits related to plant canopy, such as height and width, are closely linked to the forage and above-ground biomass yield [15]. Thus, reproductive tillers contribute to the preservation of genetic variability in future generations, while vegetative tillers harness environmental resources and provide forage [16,17].

Plant breeding with native species emerges as a complementary technology for promoting sustainable rangeland use. Breeding locally adapted plant genotypes can enhance the forage quality and yield for livestock producers, while maintaining essential ecosystem services and local biodiversity [18,19]. Moreover, native species serve as valuable resources for restoration initiatives [20]. The first steps in breeding programs with native species involve collecting and evaluating germplasm from natural populations to identify plants with desirable traits [21]. Although species often thrive in their natural habitats due to evolutionary adaptations to specific environmental conditions [15], accurately identifying the genetic variation of agronomically relevant traits requires distinguishing genetic influences from environmental factors [22]. This approach facilitates the selection and breeding of traits that improve performance and productivity in agricultural settings.

The largest arid rangelands in South America are located in Patagonia [23]. These grasslands, dominated by *Festuca* species such as *Festuca pallescens* (St. Yves) Parodi and *Festuca gracillima* Hook. f. var. gracillima, have experienced a gradual decline over the past decade [24,25]. *Festuca pallescens* is particularly notable for its palatability, cattle preference, wide distribution, morphological variation, and ecological importance. Grass steppes dominated by this native tussock occur on deep soils in topographic areas where the exposure or altitude creates favorable water balances for their development, characteristic of Patagonia’s Mediterranean climate [26,27,28]. Its distribution ranges from the Andes foothills (where it is found in patches or mosaics within deciduous *Nothofagus* forests) to the periphery of meadows in soils with low organic matter [23]. Due to the forage and ecological importance of *F. pallescens* in rangelands, its decline is a sign of rangeland degradation [29,30,31]. Studies of *F. pallescens* across a restricted geographical scale have shown high morphological and physiological variation [32,33,34,35], making it a promising species for domestication and breeding. While domestication programs are actively targeting other semiarid grasses, such as *Trichloris crinita* (Lag.) Parodi [syn. *Leptochloa crinita* (Lag) in northern Argentine rangelands [36,37], efforts in southern regions are only beginning with *F. pallescens* [20].

In this context, the extent to which environmental variation at the regional scale contributes to genetic variation in the growth, yield, and reproductive characteristics of *F. pallescens* remains poorly understood. The objective of this study is to determine the importance of genetic and environmental factors in the phenotypic expression of growth and reproductive traits related to forage and seed production. We will evaluate ten populations of *F. pallescens* from diverse environments under common garden conditions in two locations of Patagonia over two years at two specific time points. The results will provide critical information to guide the next steps in the breeding program.

## 2. Results

### 2.1. Growth Traits

**Figure 1:**
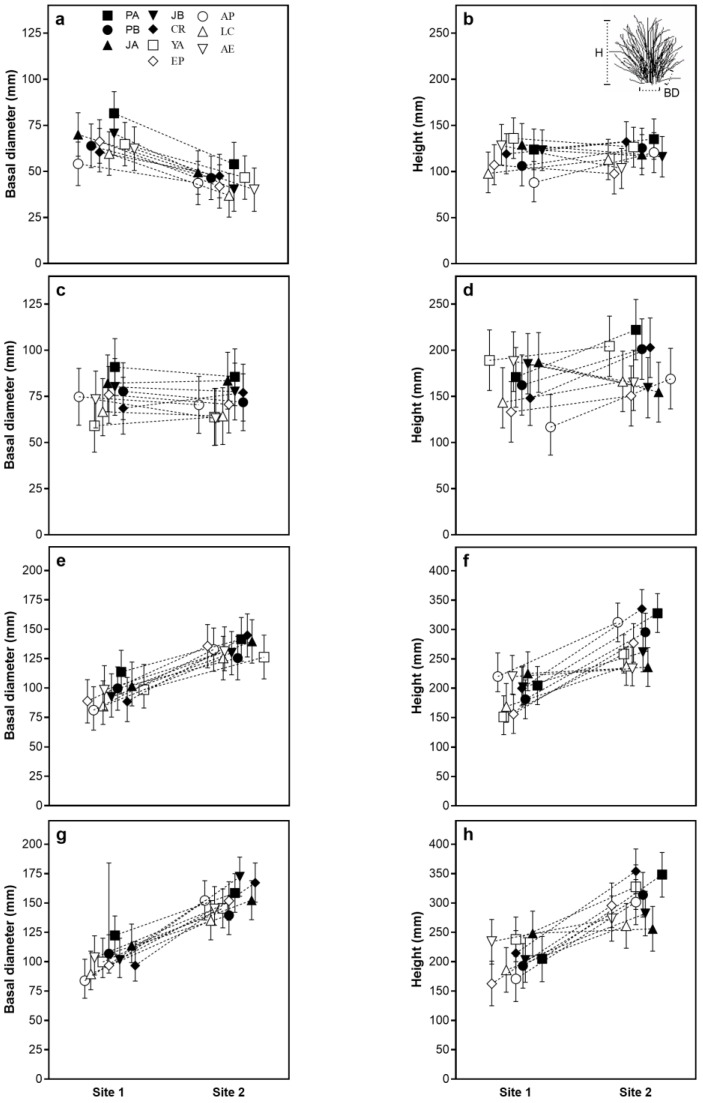
Growth traits (basal diameter and height) of the ten populations in each site, growth period, and year. Panels (**a**–**d**) show data from the first (**a**,**b**) and second (**c**,**d**) growth periods of 2018, while panels (**e**–**h**) display the same for 2019. Specifically, panels (**a**,**c**,**e**,**g**) represent basal diameter, and panels (**b**,**d**,**f**,**h**) represent height. The populations including symbol and number labels are in panel (**a**), and details about the growth measurements (basal diameter and height) are explained in the upper left of panel (**b**). Bars indicate the upper and lower limits of the confidence interval for the adjusted mean values of basal diameter and height calculated using linear mixed models for each population at each site. Sub-figure in panel b shows an illustration of the measurements.

In spring 2018, plants of the ten populations of *F. pallescens* transplanted at site 1 (EEA Bariloche) accumulated 2772.5 °Cd, while those at site 2 (CEAT) accumulated 2476.2 °Cd of thermal time. During this period, only the basal diameter showed significant differences between sites and populations. The values of the basal diameter were higher in site 1 than in site 2, showing significant differences for most populations (except for AP). By early summer 2018, the accumulated thermal time was 3450 °Cd in site 1 and 3806.8 °Cd in site 2. The values of the basal diameter for all populations were similar for both sites but the values of height increased in site 2. Differences in the values of the basal diameter were significant only among the populations, with population PA showing the highest values and populations AP and LC showing the lowest values. Interactions between the site and population were significant for height. For example, population PA showed the highest value in site 2, while population AP in site 1 and JA, JB, and AE in site 2 showed the lowest values of height (Table 1 and Figure 1).

In the spring of 2019, the accumulated thermal time was 5143.5 °Cd at site 1 and 6238 °Cd at site 2. The basal diameter values increased significantly at site 2 across all populations. Significant interactions between the site and population were observed only for height. For most populations, the height values were higher at site 2 than at site 1, except for JA and AE. By early summer 2019, the accumulated thermal time rose to 5840 °Cd at site 1 and 7028.3 °Cd at site 2. The basal diameter and height values remained higher at site 2 for all populations. Both variables exhibited significant differences between sites and populations, but the site × population interaction was significant only for height. The population with the highest basal diameter value was PA, while population LC had the lowest. For height, population PA had the highest value at site 2, while populations JA and AE had the lowest values (Table 1 and Figure 1).

The growth rate of the basal diameter was lower than that of the height in both sites. Although the interactions (S × P) were also significant, all populations grew faster in site 2 than site 1. The highest growth rate for the basal diameter was registered in population PA at site 2, and all the populations showed similar rates in site 1. The highest growth rate for height was found in populations PA and CR in site 2 and the lowest were in population EP in site 1 (Table S1).

### 2.2. Reproductive Traits

**Figure 2:**
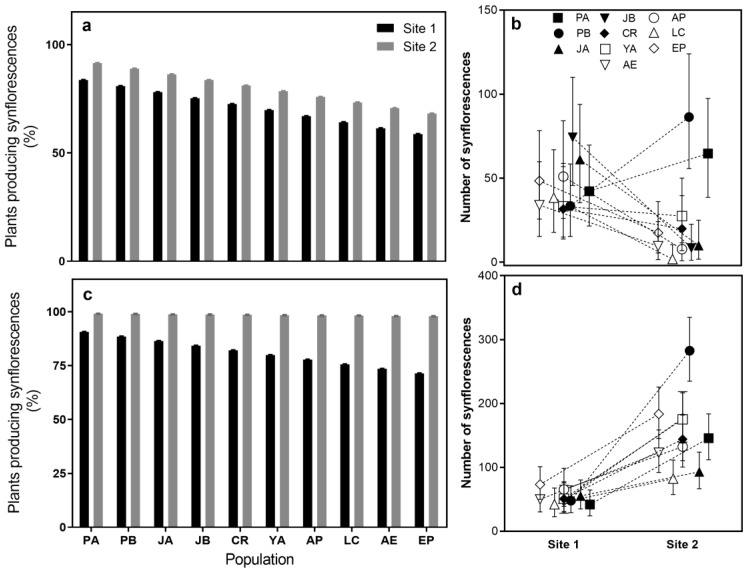
Reproductive traits of the ten populations at each site during the reproductive periods: Percentage of plants producing synflorescences in 2018 (**a**) and 2019 (**c**), and synflorescence production as the mean number of synflorescences produced by each population in 2018 (**b**) and 2019 (**d**) at each site. The bars represent the upper and lower limits of the confidence interval for the adjusted mean of each reproductive trait, calculated using linear mixed models for each population at each site.

During the reproductive period of the first growing season, the percentage of plants producing synflorescences showed a significant interaction between site and population. Populations AP and LC showed the lowest percentages of SPP in site 1 while population PB in site 2 showed the highest. In the following reproductive period, all of the populations showed a higher percentage of plants producing synflorescences in site 2 (0.95) than in site 1 (0.81), and the interaction between site and population was also significant (Table 1, Figure 2). The analysis of the synflorescence production demonstrated significant differences due to the site, population, and their interaction across both reproductive periods (Table 1, Figure 2). In 2018, populations JA and JB exhibited higher synflorescence production at site 1, whereas populations PA and PB showed the highest values for this trait at site 2. By 2019, all populations showed increased synflorescence production at site 2, with population PB being the most productive (Figure 2).

## 3. Discussion

Different patterns in the growth and reproductive traits were found in the evaluated populations of *F. pallescens*. Both the micro- and macro-environmental conditions influence the growth, development, and productivity of a pasture [38]. The temperature and water availability in which the plant species are growing regulate their morphological and physiological processes [39]. In spring 2018, the populations of *F. pallescens* showed higher values of both growth traits (basal diameter and height) and synflorescence production in site 1 compared to site 2. During this time, both sites were irrigated, so the difference in the accumulation of growing degree days (300° Cd higher in site 1) could explain why the populations of *F. pallescens* grew more in site 1 than in site 2. This pattern slightly changed by early summer, but in the spring and early summer of the following season (2019), most populations of the species showed higher values of basal diameter, height, and synflorescence production in site 2. In addition, the growth rate of the basal diameter and height was higher in site 2 than in site 1. These traits are valuable for selecting the biomass yield in dominant species of the Patagonian region [40] and other native forage grasses [37]. For example, *T. crinita* showed broad variation among natural populations in the same morphological characters that we evaluated and a positive correlation with biomass production [41].

Most of the evaluated populations inhabit sub-humid to xeric environments with a mean annual precipitation below 600 mm and variable pedo-environmental conditions [42]. The two sites where the populations were tested exhibited better micro-environmental conditions for the populations’ development than most of their maternal environments (Table 1). However, site 2 is a cultivated area largely enriched with fertilizers for agricultural and ornamental species, with higher levels of organic matter, nutrients (P and K), and possibly allophanes (non-crystalline minerals) that provide high water retention capacity [43]. Noteworthy, more than 80% of the plants producing synflorescences were registered in the second reproductive period in site 2. Altogether, site 2 was a more suitable location for maximizing both the seed and forage production of the species.

In some species of *Festuca*, the growth-related traits, such as plant size, are very plastic, while the reproductive traits show strong differentiation between populations [44]. After two years of field evaluation, the populations mostly displayed plastic patterns for growth traits (such as the basal diameter and height) and reproductive traits (synflorescence production) as they increased in either trait in site 2 (i.e., populations CR, YA, EP). Moreover, plastic patterns were recorded for almost all the populations in their reproductive traits; for example, some plants from population PB produced more than 1000 synflorescences in site 2. This is a common response in semiarid grass species to resource-enrichment pulses [45].

Previous studies on *F. pallescens* have reported variations in the species morphology across both regional and local environmental gradients, shrub coverture/vicinity, and aridity [33,46,47,48]. In addition, phenotypic plasticity has been reported for populations of this species from xeric environments [48]. While growth traits are known to show high plasticity and are generally less affected by maternal environments [44], our findings reveal a similar plasticity pattern in reproductive traits. High plasticity in both traits enables plants to adjust to changing environmental conditions, in this case, the soil type and moisture. In wild species, the energetic and resource costs of maintaining plasticity are offset by the advantage of greater ecological tolerance, consistent with a jack-of-all-trades strategy [49]. However, the extent to which these traits’ plasticity is adaptive remains unclear.

On the other hand, some populations, such as population LC, PB, and JA, maintained a compact tussock morphotype, as similar values were recorded for growth traits across both sites. It is possible that these populations produce many tillers with short leaves, which may serve as an adaptation to reduce exposure to desiccating agents [33]. However, only populations LC and JA exhibited a conservative pattern in reproductive traits, as they did not increase synflorescence production at site 2. Although population LC originates from xeric environments, similar to population AP, other factors might have influenced the growth and reproductive patterns. In this case, the topography or historical grazing pressures may have promoted the development of compact tussock morphotypes as local adaptations. Grazing can drive changes in the structure of individual plants of forage species of the Patagonian steppes. For instance, populations of *Poa ligularis* display greater size heterogeneity under moderate grazing, while intensive grazing leads to the predominance of very small individuals [40]. Moreover, intensive grazing reduces genetic variability in forage species, as the remaining vegetation patches tend to share the same genotype [50]. In contrast, non-palatable species are less affected by grazing pressure [51]. On the other hand, the original environment of population JA is located at the highest elevation, where a compact growth form may provide protection against desiccating agents [33].

### Implications for the Selection of Breeding Materials

**Figure 3:**
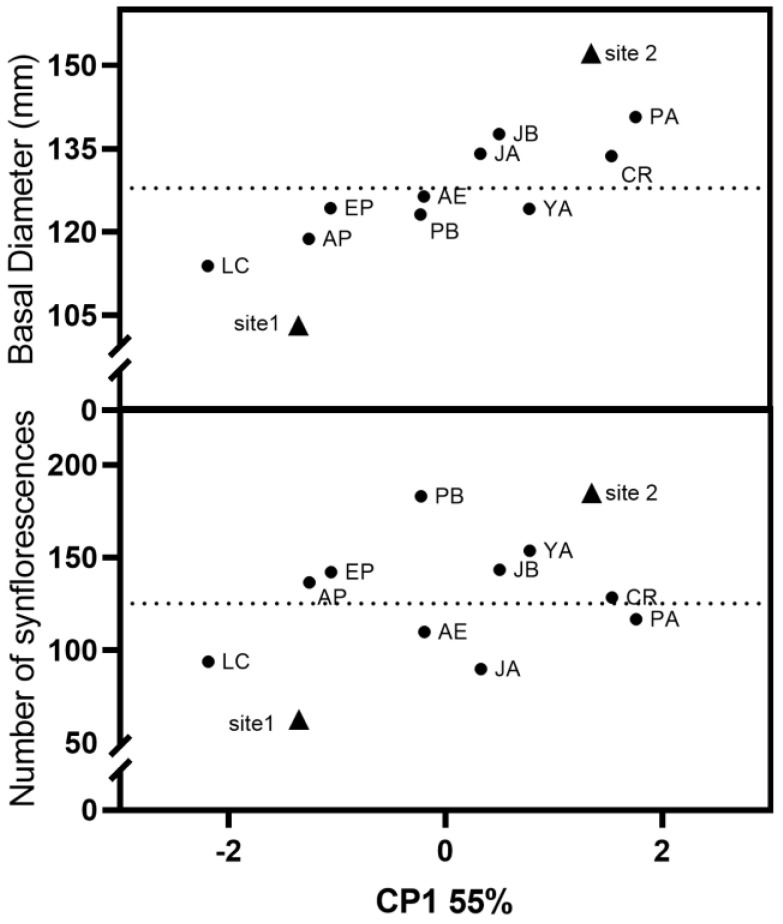
Graphical interpretation of the variation in basal diameter and synflorescence production at the end of the evaluated period across sites and populations using Multidimensional Scaling. Dotted lines indicate the average values for each variable.

At the early stages of any breeding program for forage species, two key features should be prioritized: forage yield and seed production. Populations with larger plants contribute to higher forage yields, while seeds are an invaluable genetic resource and the main tool we can offer to agricultural producers. Notably, all populations registered over 80% of plants as producing synflorescences in the second reproductive period and at both sites. In our pursuit of developing a future cultivar suited for challenging environments, primary selection should focus on populations that exhibit plasticity in both growth and reproductive traits. Consequently, populations exhibiting low growth and reproductive traits should be avoided. This led us to discard populations JA, LC, AP, AE, and EP, as they showed average to low values for basal diameter, height, and synflorescence production across both sites after two years of evaluation (Figure 3). Under fertile conditions, these populations demonstrated low to moderate productivity, indicative of a slow-growing strategy that can be advantageous for survival in stressful, nutrient-poor environments [52]. These populations, particularly LC and AP, may represent valuable germplasm for restoration efforts.

Conversely, populations JB, CR, PA, and YA exhibited moderate to high values in both growth and reproductive traits. Overall, populations JB, CR, and YA demonstrated a plastic pattern for both traits, while PA displayed plasticity only in growth traits (Figure 3). Population PB is interesting in terms of its seed production but showed a conservative growth pattern. Therefore, populations with above-average values in both basal diameter (a variable strongly associated with forage production) and synflorescence production (a variable linked to seed production) that also exhibit phenotypic plasticity (the ability to modify growth and reproductive responsiveness in diverse or heterogeneous environments) are candidates for evaluation in progeny trials to assess the heritability of these traits. As a result, the populations PA, CR, JB, and YA were selected to contribute to the development of robust cultivars capable of thriving in variable environments. Ultimately, this will support sustainable agricultural practices and restoration initiatives in diverse ecosystems.

## 4. Materials and Methods

### 4.1. Plant Material

We collected seeds from plants spaced 25 m apart following ISTA recommendations to minimize the probability of sampling clones, from 10 populations located in different environments across the species’ natural distribution (Table 2). These seeds were used to produce plants in greenhouses. Germplasm collection was conducted with authorization from the provinces of Río Negro and Chubut, in compliance with the Nagoya Protocol. Seeds were sown in 250 cm^3^ plastic cups under greenhouse conditions, using a substrate composed of organic soil, peat, and volcanic ash in a 1:1:2 ratio (containing 0.12% nitrogen, 0.06% phosphorus, and 0.09% potassium). The plants were fertilized using a soluble fertilizer, Hakaphos^®^. Once the root systems fully occupied the containers, the seedlings of *F. pallescens* were transplanted to a multi-environment genetic trial in two sites (site 1: October 2017 and site 2: January 2018). All populations of the species were identified as *F. pallescens* based on previous nuclear and chloroplast molecular analyses (ITS and *trn*L-F markers) as well as morpho-anatomical analyses [53,54].

### 4.2. Experimental Design

We established a multi-environment genetic trial in two sites. At each site, 60 six-month-old plants from each population were arranged in plots of 20 plants each, following a randomized complete block design with three blocks (N = 600 plants per site). The plants were planted 0.5 × 0.5 m apart.

**Figure 4:**
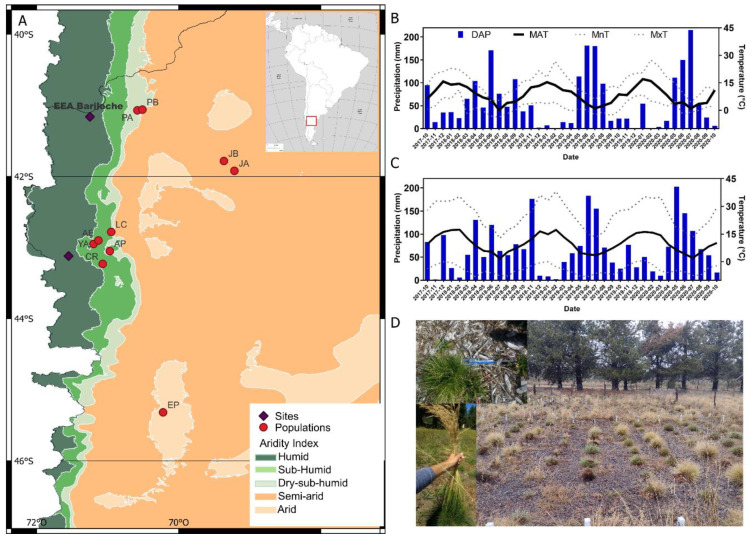
Maps showing the locations of the sampled populations and sites (**A**). Daily accumulated precipitation (DAP) and mean annual temperature (MAT) from 2017 to 2020 in site 1 (EEA Bariloche, **B**) and site 2 (CEAT, **C**) during the period of experimentation for both locations. Black lines indicate mean annual temperature, with upper and lower dotted lines representing mean maximum (MxT) and minimum (MnT) temperatures, respectively (right *y*-axis). The blue discontinuous line represents accumulated precipitation (DAP; left *y*-axis). Data provided by the Centre of Meteorological Information, National Meteorological Service, Defence Ministry, Argentina. Images illustrating the trial and basal diameter measurement using a digital caliper (top left), as well as synflorescence production (bottom left) at site 1 (**D**).

Site 1 was located at the INTA Bariloche Experimental Station (EEA Bariloche; 41°10′ S, 71°15′ W; 790 m a.s.l.), while site 2 was situated at the INTA Trevelin Agroforestry Field Station (CEAT; 43°07′ S, 71°32′ W; 350 m a.s.l.) (Figure 4). The primary differences between the two sites are related to soil type (Mollisol at site 1 vs. Andisol at site 2) and irrigation (Table 3). Growing offspring from natural populations under common garden conditions helps isolate these effects, enabling researchers to phenotypically assess traits of agronomic relevance without the confounding influences of natural environments [56]. In addition, evaluating offspring across multiple environments provides insights into how genetic material responds to varying environmental factors, such as soil type and climate [57].

### 4.3. Growth and Reproductive Periods

The vegetative growth of *F. pallescens* occurs in spring–summer, with maximum growth between October and December, when soil water availability and thermal conditions are still favorable. Meanwhile, the minimum growth rates occur between February and March, when soil water availability is very low. In autumn (April–May), there is a second growth pulse, with the beginning of the rainy season [8]. At this stage, resources are allocated to root production. The state of dormancy (or pseudo-dormancy in grasses) occurs in winter, due to low temperatures [59]. The reproductive period starts in October with the appearance of floral tillers and ends with seed dissemination mainly in December–January.

### 4.4. Growth Traits Measurements 

To assess growth traits, we measured the basal diameter of the tussock crown and the height of individual plants at two specific time points: one in spring (September) and the other in early summer (December), over two consecutive years (2018 and 2019). These time points correspond to the phases of initial vegetative growth and flowering, respectively. Basal diameter and height were measured with a digital caliper. These traits are strongly related to the individual plant biomass of dominant species in the Patagonian region [40]. Tussocks grow asymmetrically, so two perpendicular measurements of diameter for each tussock were taken [60]. We then calculated the average of these two values. We expressed the growing periods in growing degree units, using thermal time (TT) [61]. For the calculation of TT, expressed in cumulative degree-days (°Cd) since seedling transplant, we applied the following Equation (1):

(1) $$TT=\sum \begin{array}{c} \left((Tmax+Tmin)/2)-Tb\right).d \end{array}$$

where *Tmax* and *Tmin* represent the daily maximum and minimum air temperature, respectively, and *Tb* is the base temperature [62]. In this study, 0 °C was used as the Tb [63]. The growth rate (GR) was calculated by the change in basal diameter (ΔBD) or height (ΔH) related to the change in growing degree-days (ΔGGD) over the study period for each site and population (2):

(2) $$GR=\frac{\Delta BD\;or\;\Delta H}{\Delta GGD}$$

where ΔBD/ΔH represents the difference in either basal diameter or height (mm) between the end (early summer 2019) and the beginning (spring 2018) of the trial, while ΔGGD represents the total growing degree-days accumulated (°Cd) during this period.

### 4.5. Reproductive Traits Measurements

We registered the plants producing synflorescences (SPP), categorizing them as either producing synflorescences (SPP = 1) or not (SPP = 0) during the reproductive period. Additionally, we counted the number of synflorescences to obtain the synflorescence production (Sy) for each plant in both years. Synflorescences were harvested at the end of each reproductive period, that is, January–February in site 1 and December–January in site 2.

### 4.6. Data Analyses

We analyzed the variation in each trait using the following linear mixed effects model:

(3) $$Y_{ijkl}=S_{i}+P_{J}+{\left(S\;x\;P\right)}_{ij}+{B\left(S\right)}_{ki\;}+e_{ijk}+d_{ijkl}\;$$

where S is the fixed effect of site (i = 1,2); P is the fixed effect of the population (j = 1,…,10); (S × P) is the interaction effect between site and population; and B is the random block effect (k = 1,...,3) nested in S_i_, with *e*_ijk_ and *d*_ijkl_ as experimental and subsampling errors, respectively. Most variables displayed a Gaussian distribution; however, synflorescence production required a square root transformation. All the variance analyses were run in RStudio 2024.4.1.748 [64], with the lmer package [65]. For the variables with Gaussian distribution, we used REML, while the Laplace approximation to the marginal likelihood was used for the binary data.

Finally, we used Multidimensional Scaling (MDS) to create visual representations of the relationships between populations and sites, and then constructed a scatter plot with the dimension that explained 55% of the variation and the mean values of basal diameter and synflorescence production from early summer 2019 for each population and site. In this plot, sites or populations that are similar are positioned closer together, while those that are more dissimilar are placed farther apart. This visualization not only reveals patterns among sites and populations but also allows the association of these patterns with the average values of each variable. MDS was carried out using NavureTeam 2023 [66], while all graphs were elaborated with Prism version 8.0.0 for Windows (GraphPad Software, San Diego, CA, USA).

## 5. Conclusions

The tested environments provide empirical evidence of species performance. By isolating the effects of the source environments, we can attribute the phenotypic differentiation in both the growth and reproductive traits to genetic factors. Most of the populations displayed opportunistic patterns in their growth and reproductive traits in response to micro-environmental cues. Further studies will confirm these patterns as forage quality is being evaluated and C:N ratio would provide information on their position in the plant economic spectrum. These findings facilitated the selection of populations with optimal performance in plant growth and seed production. At the end of the second growing period, most of the populations performed better at site 2, suggesting more favorable environmental conditions for the species. In addition, over 80% of the plants in all the populations produced seeds, which are promising results for both restoration and breeding efforts. Our results show that this set of *F. pallescens* populations is quite sensitive to environmental conditions but maintains consistency in relative performance. Finally, the standout populations were selected for progeny trials, marking the next step in the breeding program and shortening the path towards a synthetic Patagonian grass.

## Figures and Tables

**Table 1:** Statistics associated with fixed effects (S: site, P: population, and S × P: the interaction between them) of mixed-effects models analyzing variations in growth and reproductive traits. Table entries are F values using Satterthwaite’s method and X^2^ values for synflorescence-producing plants. * indicates significance with *p* < 0.05.

Year	2018						2019						2018–2019		
Growth Period	Spring			Early Summer			Spring			Early Summer			Growth Rate		
	Main Effects			Main Effects			Main Effects			Main Effects			Main Effects		
Growth traits	S	P	S × P	S	P	S × P	S	P	S × P	S	P	S × P	S	P	S × P
	df = 1	df = 9	df = 18	df = 1	df = 9	df = 18	df = 1	df = 9	df = 18	df = 1	df = 9	df = 18	df = 1	df = 9	df = 18)
Basal diameter	56.37 *	1.85 *	0.54	0.29	2.27 *	0.24	53.72 *	0.99	0.67	125.94 *	2.30 *	1.58	50.43 *	0.71	1.98
Height	0.24	1.87	1.35	2.03	3.45 *	2.54 *	126.7 *	4.48 *	3.88 *	72.96 *	2.92 *	3.30 *	24.67 *	2.81 *	4.9 *
				Harvest 2018–2019						Harvest 2019–2020					
				Main effects						Main effects					
Reproductive traits				S	P	S × P				S	P	S × P			
				df = 1	df = 9	df = 18				df = 1	df = 9	df = 18			
Synflorescence production				15.34 *	3.4 *	5.86 *				131.14 *	5.23 *	4.69 *			
Plants producing synflorescences				4.75	6.54 *	0.83				0.98	4.71 *	3.69			

**Table 2:** Environmental characterization of ten populations of *Festuca pallescens*. Floristic physiognomic type (FPT) was characterized according to [55]). Lat: latitude; *Long*: longitude; Alt: altitude (m a.s.l.); Pp: precipitation (mm). RN: Río Negro, CH, Chubut, Argentina.

ID	Population	Lat	Long	Alt	Pp	FPT
PA	Experimental Field Pilcaniyeu, RN	−41.06	−70.52	1260	264	Shrub-grass steppe
PB	Experimental Field Pilcaniyeu RN	−41.07	−70.58	970	264	Meadow
JA	Ing. Jacobacci, RN	−41.92	−69.22	1400	170	Grass steppe
JB	Ing. Jacobacci, RN	−41.92	−69.22	970	170	Salty Meadow
CR	Cronómetro, CH	−41.23	−71.07	875	500	Shrub grass steppe
YA	Yagüe, CH	−42.95	−71.20	748	450	Shrub-grass steppe
AP	A. Pescado, CH	−43.03	−70.97	679	350	Shrub-grass steppe
LC	La Cancha, CH	−42.78	−70.95	778	300	Shrub-grass steppe
AE	Aeropuerto, CH	−42.90	−71.13	800	500	Shrub-grass steppe
EP	El Puesto, CH.	−45.32	−70.22	493	150	Meadow

**Table 3:** Environmental characterization of each site and conditions during the period of evaluation. Temperature and precipitation are expressed in degrees Celsius (°C) and millimeters (mm), respectively. Soil analyses for each site included Electrical Conductivity (E.C., decisiemens per meter), organic matter (O.M., percentage), nitrogen (N, percentage), phosphorus (P, parts per million), and potassium (K, parts per million).

Environmental Conditions	Site 1	Site 2
Mean annual temperature	8.6	10
Mean minimum temperature	3.5	4.9
Mean maximum temperature	13.8	15.1
Mean annual precipitation	840	976
Soil type	Mollisol	Andisol *
pH	7.25	6.09
E.C.	0.04	0.105
O.M.	4.3	6.1
N	0.25	0.185
P	2.14	42
K	342	807
During the trial	site 1	site 2
Mean annual temperature	9.4	10.41
Mean accumulated precipitation	62.3	71
Irrigation	21.9 m^3^/ha three times a week, sprinkler	10.9 m^3^/ha every day, drip
Irrigation period	2017–2019	2018–2020

* based on [58].

## Data Availability

The raw data supporting the conclusions of this article will be made available by the authors on request. The data are not publicly available due to an ongoing study and further analyses are in progress.

## Supplementary Materials

The following supporting information can be downloaded at https://www.mdpi.com/article/10.3390/plants14050736/s1: Table S1: Growth features of the ten populations of *Festuca pallescens* for early summer 2018 and spring 2019 in each site.
