# Supplementary material for: Genetic Variation of Growth Traits and Seed Production in a Patagonian Native Pasture in Semiarid Rangelands Under Different Environmental Settings
Source: Plants (Basel). 2025 Feb 27;14(5):736. doi: 10.3390/plants14050736 (PMC11901679; doi:10.3390/plants14050736)
Supplement: Supplementary file 1 [file plants-14-00736-s001.zip › plants-3445370-supplementary.pdf]

Supplementary material

Table S1: Growth features of the ten populations of *Festuca pallescens* for Early Summer 2018 and Spring 2019 in each site. Growth traits correspond to basal diameter (BD, mm) and height (H, mm), reproductive traits are the proportion of seed producing plants (SPP) and synflorescences production (Sy, n°). Adjusted mean values of BD, H and Sy were obtained from a general linear mixed effects model while SPP values were calculated with a generalized linear mixed effects model. Growth rate (mm/°Cd) are indicated for each growth trait. LCL and UCL are the low and upper confidence limits for each estimated value.

		Spring 2019 (2772.5°Cd)		*Early summer 2018 (3450 °Cd)*				*Spring 2019 (5143.5 °Cd)*	
	**Population**	**BD (mm)**	**H (mm)**	**BD (mm)**	**H (mm)**	**SPP**	**Sy (n°)**	**BD (mm)**	**H (mm)**
**site 1**	PA	81.5±18.2	123.8±35.1	90.9±20	170.9±51.8	0.87	51±41.2	113.6±23.2	204.5±54.2
	PB	63.9±20.5	106±30.9	77.8±22.9	162.2±46.5	0.82	41.1±36.5	99.6±28.8	181.1±68.4
	JA	70±24.1	129.7±47.8	82.1±26.2	186.9±59.3	0.78	73.8±57.2	103.4±25.5	229.2±70.8
	JB	70.5±17.9	123±37.3	80.1±20.8	185.2±58	0.87	82.4±45	94±16.2	204.9±52.5
	CR	61.4±22.8	119.1±40.1	69.8±23.3	150.4±63.3	0.65	41.4±42.5	89.9±25.7	203.2±70.5
	YA	64.8±22	136±45.5	74.8±22.9	189±64.5	0.72	40.9±37.6	101.9±31.6	227.6±73.3
	AP	54.1±31.2	88.9±60	60.1±31.4	118.7±70.8	0.52	74±78.5	82.6±35.8	153.7±84.8
	LC	59.7±22.3	98.9±37.5	69±26.8	148.4±57.5	0.48	53.7±62.7	87.5±27.5	174.8±67.6
	AE	62.3±21.2	128.7±48.2	73.3±23.8	187.9±63.3	0.67	40.9±37	99.9±24	223.4±70.6
	EP	66.2±19.5	107.5±39.2	75.8±21.5	133±48.8	0.75	60±49.1	88.9±23.2	155.8±58.7
		Spring 2019 (2476,2°Cd)		*Early summer 2018 (3676.4 °Cd)*				*Spring 2019 (6238 °Cd)*	
	**Population**	**BD (mm)**	**H (mm)**	**BD (mm)**	**H (mm)**	**SPP**	**Sy (n°)**	**BD (mm)**	**H (mm)**
**site 2**	PA	53.9±14.8	135±33	85.3±18.9	222.2±48.7	0.95	81.1±69.7	141.4±34.8	327.7±74.9
	PB	46.5±16	125.3±29.1	71.8±19	201.1±38.2	1	104.1±86.5	125.4±21.7	295.5±47.1
	JA	49.5±12.8	118.3±21	83.5±14.9	154.6±27.8	0.82	15.7±19.2	139.7±29.3	236±56.5
	JB	40.2±15.2	115.9±25.4	77.6±21.5	159.3±33.5	0.75	14.5±18.4	129.8±41.9	261.3±52.6
	CR	47.5±16.2	132.2±29.5	77±24	202.8±51	0.82	34.6±46.2	144.9±41.4	335.2±73
	YA	46.7±14.8	126.6±35.9	70.4±19.5	204.3±42.3	0.90	40.4±43.9	126.3±34.6	312.3±53.8
	AP	43.7±15.2	120.6±30.7	63.8±18.2	169±48.1	0.65	15.1±21.9	132.8±29.6	258.5±64.2
	LC	37±16.1	113±29.9	64.3±22	166.1±47.3	0.45	8.1±23.7	125.5±41.8	237.5±60.7
	AE	40±16.2	103.5±27.5	64±20.1	167.5±40.8	0.77	17.8±33.9	133.2±29.7	237.3±59.9
	EP	41.8±14.9	97.5±20.2	70.6±15.1	150.5±37.7	0.88	24.8±26.7	135.7±24.5	277±60.1
